# Quantitative analysis of mass mortality events in salmon aquaculture shows increasing scale of fish loss events around the world

**DOI:** 10.1038/s41598-024-54033-9

**Published:** 2024-03-07

**Authors:** Gerald G. Singh, Zaman Sajid, Charles Mather

**Affiliations:** 1 Ocean Nexus, School of Environmental Studies, University of Victoria, Victoria, Canada; 2 Mary Kay O’Connor Process Safety Center, Texas A&M University, College Station, USA; 3 Department of Geography, Memorial University of Newfoundland and Labrador, St. John’s, Canada

**Keywords:** Environmental impact, Ocean sciences, Sustainability

## Abstract

Globally, salmon aquaculture promises to contribute to sustainable sources of animal protein for a growing human population. However, the growth of the industry also includes increased reports of mass mortality events—disaster events where large numbers of fish die in short periods of time. As salmon production increases in scale and more technology is used to grow salmon in contexts otherwise not suited for them, there is a possibility for more frequent and more severe mortality events. Despite investigations into specific cases of mass mortality events—no global study has been conducted to see if large scale mortality is increasing in frequency and scale. Using a global dataset of publicly available and government-collated data on salmon mortality events including nations responsible for the majority of salmon aquaculture, we document trends in mortality events, showing that in some of the major salmon producing nations of the world (in particular Norway, Canada, and the UK), mass mortality events have increased in frequency from 2012 to 2022. We also show that the scope of mass mortality events has increased over time—that is, the upper bound of how many fish were killed in a specific mortality event has increased over time. Finally, the expected maximum size of a mass mortality event differs from country to country, but is likely much larger than site and jurisdictional thresholds of concern for animal welfare, early warning thresholds, and capacity to respond to mortality events. The consequences of the increased scale and scope of mass mortality events extend past aquaculture production to include severe consequences to aquaculture companies and to coastal communities who depend on aquaculture. Our results agree with predictions of the concept of “manufactured risk”, which suggests that risk emerges from the aggressive use of technology to optimize production in variable environments, and we argue that there is a need for more fine-scale and standard data collection on salmon mortality events, and that future investigations into salmon aquaculture should increase focus on disaster potential and realization.

## Introduction

The global salmon aquaculture industry is seen as a sector that has the potential to produce a sustainable animal protein for the planet’s growing population^1,2^. Indeed, representative organizations of the aquaculture industry claim that farmed salmon is a ‘climate friendly’ protein based on its carbon footprint and low greenhouse gas emissions relative to other animal proteins^3^. At the same time, the sector is facing the problem of mass mortality events (MMEs)—events where large numbers of farmed fish die in a short period of time. While fish mortality has always been a concern for farmed salmon production^4–9^, MMEs are of particular concern because of the scale and rapidity of loss, and associated effects to communities dependent on aquaculture. MMEs have been recorded in most of the major salmon producing countries including Norway^11^, Canada^12,13^, Scotland^14^, Ireland^15^, Chile^16,17^, the United States^18^, and Australasia^19^. Analysis of MMEs, often led by national regulatory authorities, tend to focus on the causes of single events that involve one or several production sites with a view to mitigating future MMEs^11,15^. These studies are useful in providing in-depth analysis of the magnitude and possible causes of single mass die off events and report changes in mortality over time, but few if any studies have attempted to quantitatively assess trends in extreme loss events—MMEs—at multinational to global scales. Here we ask: is the scale and magnitude of MMEs in salmon aquaculture increasing in scope and over time?

The salmon aquaculture sector has grown very rapidly since it was first established in Norway in the 1960s^20^. Production has spread from Norway to other European countries as well as to Chile, Canada, the United States and Tasmania making it one of the fastest growing food production systems in the world. While production has increased dramatically in the last 60 years on the basis of a highly successful and standardized production model involving cages located in near shore ocean sites^21^, the industry faces considerable environmental challenges, including that environmental stressors (some amplified by climate change) affect aquaculture production and salmon health and that production affects the wider environment and wild salmon^11^. Warming oceans have posed a significant challenge to salmon aquaculture and this warming has exacerbated problems of rise in water temperature and hypoxia (low oxygen levels) contributing to MMEs^22^. Additional environmental problems include existing and new diseases, sea lice, water quality problems, and harmful algae blooms^23^. Living fish expel their wastes into water as a result of their bodily function while the decomposition of dead fish releases nutrients in the water, which causes algae blooms—making survival difficult for the remaining fish. In MMEs, these challenges often appear to work in tandem: higher temperatures may lead to hypoxia, which in turn can be fatal for fish that are immune compromised due to disease^22^.

The risks associated with salmon production that lead to MMEs are, however, rarely only environmental. Mortalities including those that result in MMEs are often caused by a combination of natural events and human decisions. For example, salmon mortality within aquaculture production facilities can often be the result of production practices such as mechanical and thermal delousing that coincide with other environmental and physiological conditions compromising fish health^6^. Similarly, overuse of antibiotics and antiparasitics can cause bacteria and parasites to develop resistance to them, and these treatments can become ineffective, which leads to an increased risk of MMEs^23^. Aquaculture operations manufacture systems where high densities of salmon allow for large populations to face mortality-inducing conditions simultaneously. The consequences of these MMEs are not limited to the stock of salmon but can have significant impacts to the surrounding environment (through nutrient release and the creation of anoxic “dead zones”) and the people working in the aquaculture production facilities^24^, and the consequences tend to worsen with increased magnitude of MMEs. For example, MMEs can be met with regulation that strips a company’s permit to raise fish, which can devastate local economies, e.g.^11^. The process of collecting and disposing of large volumes of dead fish may also have potential occupational health and safety consequences for workers involved in these labour-intensive and potentially risky tasks^24,25^.

The research in science and technology studies refers to risks that come about from human development and from human infrastructure, rather than external impacts to human communities alone, as “manufactured risk”^26^. Manufactured risk occurs when human decisions and infrastructure create or enhance contexts for consequential events. Manufactured risks are frequently the outcome of industrialization and modernization, in which technology and procedures are developed to boost efficiency and output but can also represent dangers if not managed appropriately or increase vulnerability to disasters by exposing a system to greater environmental variation that serve as hazards^27^. To our understanding, manufactured risk has yet to be quantitatively explored in aquatic food systems. Aquaculture, as an engineered system set up to optimize food production within an increasingly uncertain and variable environment, raises questions on whether risk is being manufactured in this food system. New efforts to expand aquaculture production under climate change and to gain maximum benefits from introducing salmon aquaculture to new environments (such as growing interest to grow salmon aquaculture offshore) present new potentials for manufactured risk, cf.^28^. Our research explores whether the frequency and scale of MMEs in salmon aquaculture is increasing, which is the first step in understanding if trends in salmon aquaculture are increasingly introducing manufactured risk—an important concern in a food system we may come to rely on more in the future.

## Results

**Figure 1:**
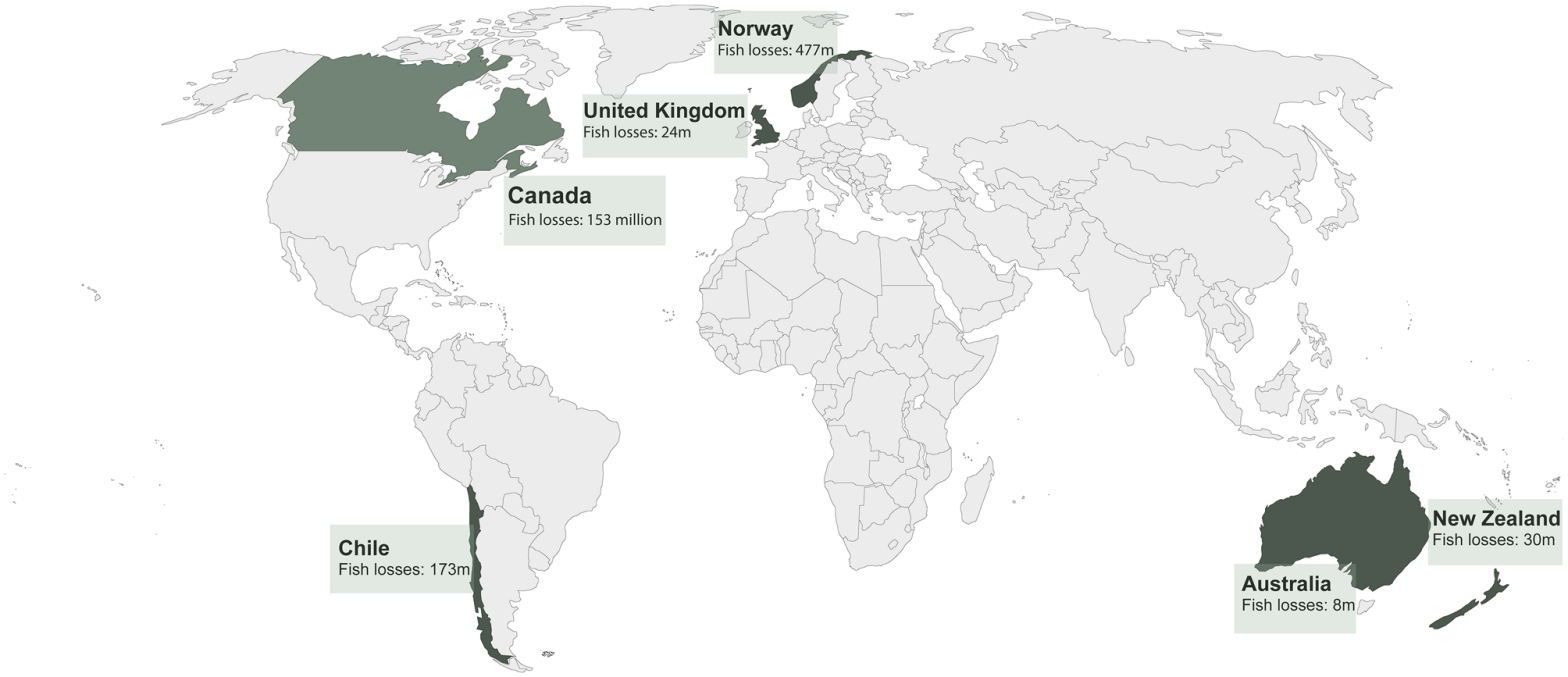
The scales of fish lost in salmon aquaculture around the world during the period 2012–2022 from collected data (see “Methods” and Supplement). The map was created by the authors using Adobe Illustrator—Version 24.2.2 (adobe.com).

We found salmon mortality records for the top 4 salmon aquaculture producing nations of the world (Norway, Chile, United Kingdom, and Canada) which in 2021 cumulatively produced approximately 90% of global salmon aquaculture output^29^. We also found MME records for Australia (the sixth largest producer) and New Zealand (the tenth largest producer), and cumulatively these six nations produced over 92% of the world’s salmon (by live weight) in 2021^27^. Our database records mortality of 865,000,000 fish in these six nations over the last decade (Fig. 1, Supplementary Fig. 1 shows the records of loss events over time per country in a dynamic map).

**Figure 2:**
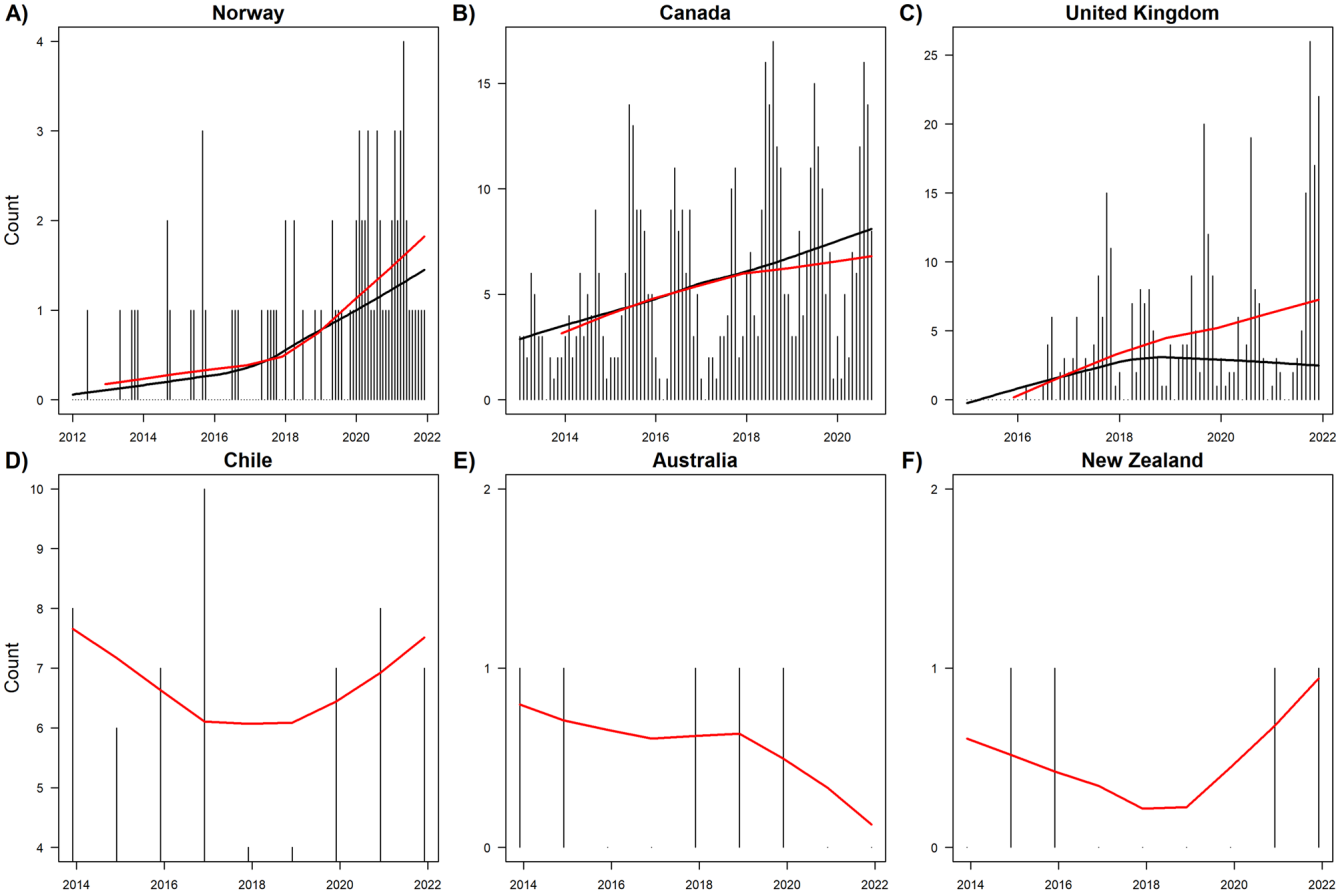
Trends in the counts of MMEs across countries. For (**A**–**C**), events are counted as the top 10% of events as measured by the number of fish lost, within each country. For (**D**–**F**), events are counted as measured by the top 50% events, since there are too few observations to track the top 10%. (**D**–**F**) should be considered illustrative and not conclusive given the aggregation of data and low samples. Trend lines represent monthly (black) and yearly (red) lowess regression across the counts of events.

We found that the frequency of the highest mortality events (as defined by the top 10% of highest mortality events from 2012–2022 within each country) increased over time for Norway, Canada, and the United Kingdom (Fig. 2). Trend analysis showed significant monotonic trends over time for Norway (Kendall’s tau = 0.961, p = 0.0248), Canada (Kendall’s tau = 0.3, p = 0.0177), and the United Kingdom (Kendall’s tau = 0.457, p = 0.000854). For Chile, Australia, and New Zealand, no clear trends in the frequency of the top 50% highest mortality events were observed, though we note that there was considerably less data to analyze due to aggregated data reporting in these countries.

**Figure 3:**
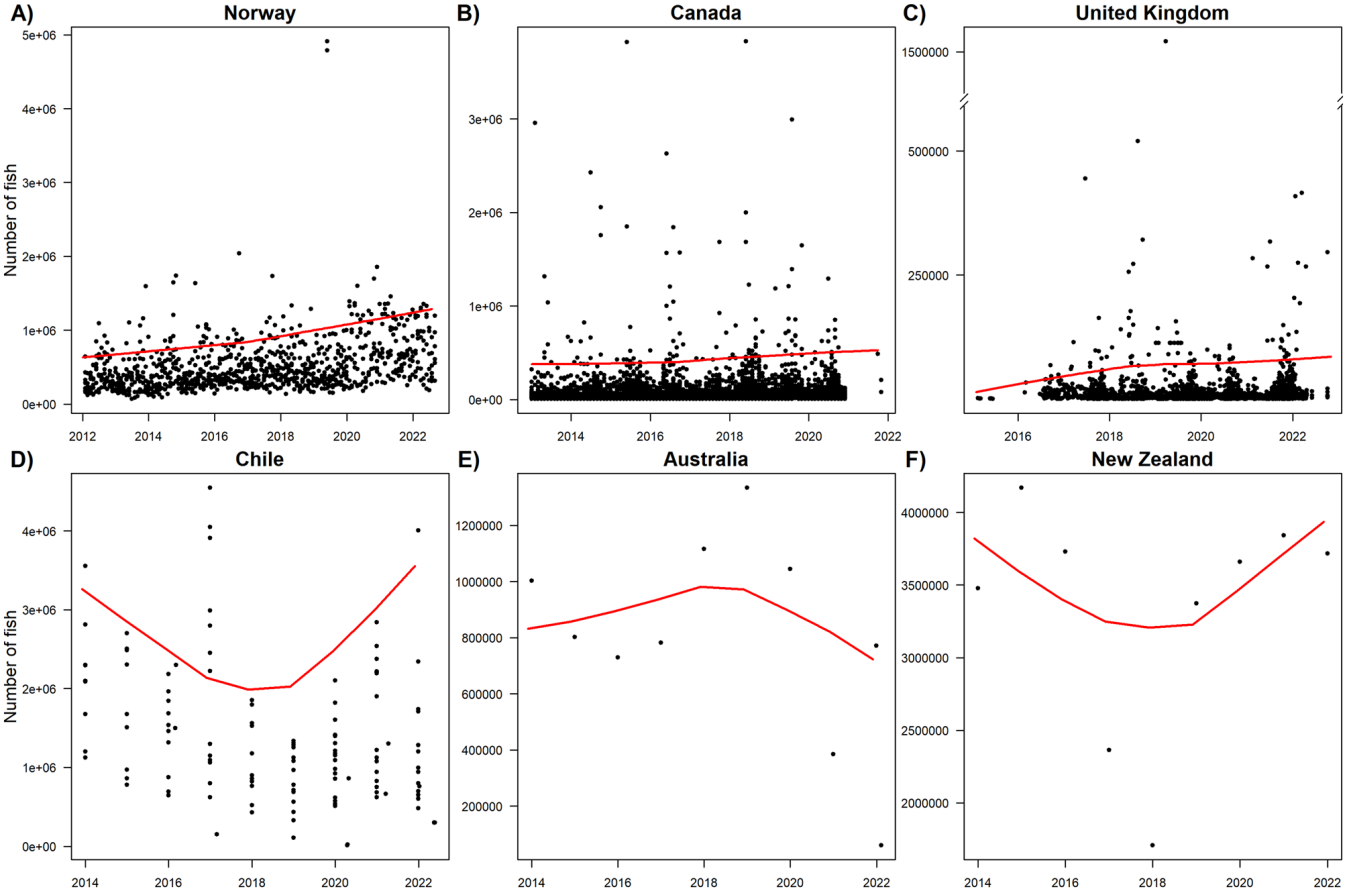
Trends in the scale of salmon mortality events over time. The continuous red line represents a local polynomial regression (lowess regression) line following the maximum of monthly events in (**A**–**C**), and follows the maximum of yearly reportings in (**D**–**F**). Note the break in the y-axis for (**C**).

Trends in the sizes of the largest magnitude mortality events shows that the scale of MMEs have grown over time for Norway, Canada, and the United Kingdom, reflected in an increasing scope of loss for individual MMEs (Fig. 3). Trend analysis show significant monotonic trends over time for Norway (Kendall’s tau = 0.432, p = 4.77 × 10^–7^), Canada (Kendall’s tau = 0.766, p = 2.22 × 10^–16^), and the United Kingdom (Kendall’s tau = 0.606, p = 2.22 × 10^–16^). In Norway, where data was collected at county levels instead of production site levels, the increasing trends in frequency of highest mortality events and magnitude of upper limit of loss did not co-occur with a trend in the increase in the number of production sites within Norwegian counties. That is, the number of production sites across Norway remained relatively constant, with no statistically significant trend in production sites (Supplementary Fig. 2). Trend analysis for Chile, Australia and New Zealand showed no clear trend, but data collection for these three countries is not per mortality event but for individual production facilities aggregated each year, meaning, with the available data, we cannot estimate the scale of losses of individual MMEs for these countries.

**Figure 4:**
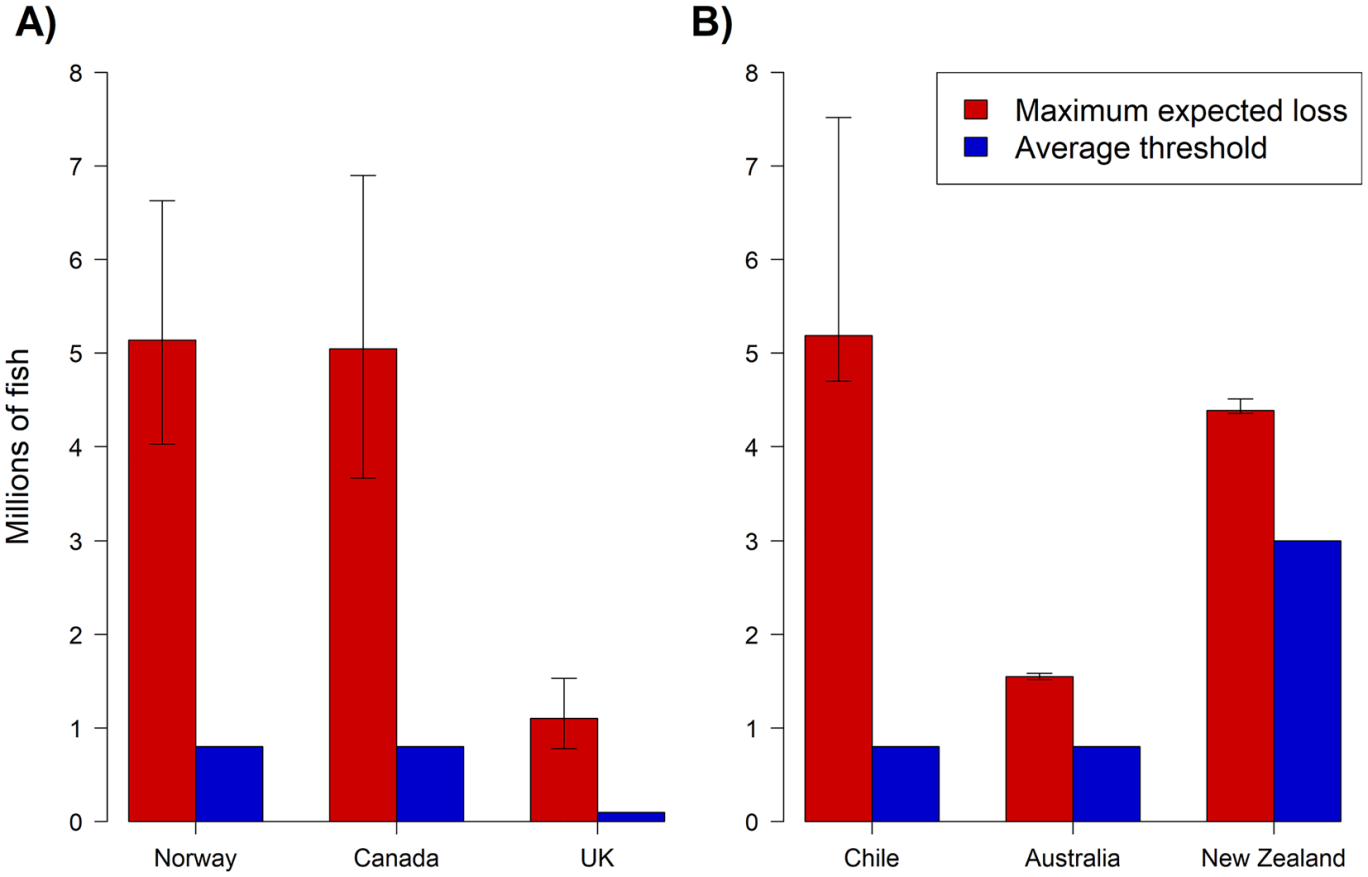
The expected maximum loss per event for Norway, Canada, and the UK (**A**) and expected maximum yearly loss per year for New Zealand, Australia, and Chile (**B**). For reference, these figures are compared against an average threshold, which reflects an average of what production sites define as MMEs. Estimates of maximum loss show the Expected Shortfall values, with error bars representing the number of fish estimated to be lost in a 1/1000 event (lower bound) and a 1/10,000 event (upper bound).

In estimating the largest potential mass die off event within Norway, Canada, and the UK (where loss data is reported per month or more frequently), we found that all countries have potentials for a single mass mortality event much higher than the average company-defined threshold. Norway has the potential for the largest loss (with an expected loss of the worst 0.1% of cases equalling 5.14 million fish), however Norway’s data is aggregated to the county scale. Among countries with data at the site scale, Canada has the largest potential for a single mass mortality event (with an expected loss of the worst 0.1% cases equalling 5.05 million fish, Fig. 4A). For estimating the potential annual loss within Chile, Australia, and New Zealand (where loss data is reported per year), we again found that all countries have potentials for loss over a single year to greatly surpass the average company-defined threshold. Chile has the highest potential loss (at 8.19 million fish), followed by New Zealand (4.39 million fish) and then Australia (1.55 million fish, Fig. 4B).

## Discussion

Over the past decade, salmon aquaculture has generally increased in terms of geographic scope, frequency, and in the scope of magnitude of individual MMEs (as documented by a growing upper bound of magnitude of mortality). While we find growth in the frequency and scope of magnitude of MMEs in Canada and the United Kingdom because they report at per-site levels, we also document a greater frequency of high mortality at county scales in Norway, and suggest that this adds an important dimension to the broad problem of fish mortality in Norway’s farmed salmon sector^10^. Simultaneously, we suggest that increasing trends of the scope of high mortality at county scales in Norway may reflect increased sizes in individual events at production sites. To justify these conclusions, we point to the findings that the increased frequency of high loss and scope of high-loss observed at county levels do not correspond with similar increases in the number of production sites. That is, higher levels of mortality were observed over time while the number of production sites remained relatively constant.

The increased frequency of high mortality events in many parts of the world may track the growth of aquaculture in terms of the number of sites and geographic spread of aquaculture production. However, the increased scope of MMEs may not be attributed to the growth of aquaculture production around the world. Instead, the growing scope of loss may be a consequence of the technologies and practices intended to increase productivity at production sites, such as technology to optimize production conditions and a greater tendency to move production sites offshore^28^.

The increase in distribution, frequency, and scope of the magnitude of MMEs adds to the growing concerns about global aquaculture’s ability to feed the future. Globally, salmon aquaculture has grown in some regions more than others, with FAO data showing that the most growth in production between 2016 and 2020 occurred in Norway and Chile, and more modest to stagnant growth in the UK, Australia, New Zealand, and Canada (Supplementary Fig. 3). There is also concern that future growth in aquaculture is optimistic, with recent research suggesting that global aquaculture has peaked and may be on the verge of decline^30^. While it is too early to suggest that MMEs may offset global production, it may add to the list of factors pointing away from aquaculture production growth.

Within each country, the expected loss of the worst 0.1% of events indicates that every major salmon producing country faces the possibility of having events that severely surpass thresholds. The thresholds may reflect an individual company or county’s ability to contain and respond to the adverse impacts of MMEs, may reflect early warning thresholds indicating a potential disease/mortality problem, or may reflect thresholds reflecting animal welfare. If the country specific thresholds represent the general capacity of companies and agencies in each country to address mortality events when they occur, then all countries have the potential to be overwhelmed by MMEs. Canada and the UK may face greater single-event consequences for MMEs compared to Norway despite Norway having greater estimated maximum loss since losses are estimated per production site within a month in Canada and the UK, and per county in a month in Norway.

If the thresholds do not reflect capacity restrictions but early-warning systems, then the expected loss of the worst 0.1% of events indicate that the worst case events often far surpass these early indicators. In many countries there are requirements for companies to have contingency plans to address dead fish, however some recent literature questions the efficacy of these plans to address the health, environmental, and social consequences of MMEs, and in some cases they were inadequate or lacking^24,25^. While we acknowledge that because of data limitations our estimates of thresholds may not account for all production sites (and therefore estimates of average thresholds may be off), in most cases we estimate threshold exceedances in the millions of fish which we suggest is alarming enough to warrant further investigation into management and contingency plans and their capacity to contain and respond to MMEs. Ignoring the thresholds, we still suggest that the potential loss of millions of fish is alarming, especially in countries where the data is reported per production site (Norway reports loss per county).

Our data collection revealed inconsistent data reporting across the world. Some countries report at site level, while others at regional (county). Some report at relatively precise temporal scales (months or less) while others at yearly. In continuing to build a database on MMEs, we recommend that countries and jurisdictions around the world standardize data collection and reporting so that the risks of production can be traced in standardized formats along with metrics that track the food production benefits.

MMEs pose risks and adverse consequences for salmon production, but the consequences of MMEs are also significant for aquaculture companies and the communities that depend on aquaculture production for employment. A recent report by a Non-Governmental Organization (NGO) has estimated the cost of salmon mortalities in four of the largest producing countries (Norway, Chile, Canada, and the UK) since 2013 to be more than 15 billion dollars^31^. In some cases, our findings of expected extreme losses show greater expected maximum losses in countries with lower production than others (e.g. New Zealand vs. Chile). Contexts where lower production countries face greater extreme losses may face greater individual impact on local economies and ecologies since they will have a greater effect on total production. The impact of MMEs in communities dependent on salmon aquaculture can be particularly devastating. In Chile in 2016 an MME at a production site in the Chiloe region caused by red tide resulted in the death of over 6 million fish, representing more than 12 per cent of annual production^32^. The economic and social costs to the Chiloe region were significant: 4,500 people directly employed by the industry lost their jobs and the livelihoods of 6,000 inshore fisherman were affected, and even the tourism sector was affected because of the environmental impact associated with disposing of dead fish^32^. The resulting economic devastation to the Chiloe region was such that it required government cash supports for affected households.

There is a significant reputational—or social licence—impact of MMEs on companies involved with aquaculture production and to the industry as a whole^24^. For example, in Scotland where recent significant challenges with mass mortalities have led to calls by environmental NGOs, groups concerned with animal welfare, and politicians to restrict the growth of the sector in Scotland in light of these challenges^33^. MMEs may also result in responses by regulators: in Canada, for example, the death of 2.5 million fish on the south coast of Newfoundland resulted in provincial regulators withdrawing the licence for the company involved^11^.

Part of the community and health impacts of MMEs have to do with the post-event clean up. An emerging area of inquiry examines the relationship between MMEs and occupational health and safety^24^. In most (if not all) jurisdictions where salmon aquaculture is regulated, MMEs require the rapid mobilization of workers to remove dead salmon from net pens and to transport them to where they can be disposed of or, in some cases, processed for human or animal consumption^24^. There is very little research on the occupational health and safety implications of these events in an industry that has relatively high rates of injury^25^.

Ironically, some of the methods employed to reduce fish loss and maximize fish production may increase the rate and scale of MMEs. For example, adopting new technologies and early warning systems as well as programs aimed at reducing the vulnerability of salmon to warming events, diseases and pest infestations through improved feeds or selective breeding, can lead to an increased sense of security and justify the growth of salmon in increasingly risky contexts^28^. New technologies, improved feed, and early warning systems are aimed at addressing some of these risks that are a consequence of operating in increasingly variable environments^34–37^. These include devices that measure temperature, water velocity, oxygen and salinity within the cage environment to remote sensing technology at larger scales that provide data on weather, currents and ocean temperatures. The more advanced systems use artificial intelligence to monitor fish behaviour using underwater cameras during feeding and to warn of potential disease outbreaks^34^. Overall these systems aim to improve decision support in a context of a rapidly changing environment for fish farming in the ocean, but since they often attempt to promote productivity and create justifications for increased production capacity into riskier locations such as offshore and high energy sites, they have the potential to expose greater amounts of fish to hazards that can generate larger MMEs.

Fish farming technologies are geared to managing and controlling production in ocean systems that are changing in trend and variation that are difficult to predict and comprehend at short time-scales, which can lead to aquaculture disasters in the form of MMEs. Scholars in the field of science, technology and society have examined a wide range of disasters and have raised a number of problems with how these events are understood and how industry and regulators respond to them^27^. First, disasters are often seen as a natural event associated with the natural environment (such as climate change or pathogens) that impact human designed production system. However, all disasters are the intersection of environmental hazards and human infrastructure and decisions^38^. In the case of aquaculture, while MMEs in salmon aquaculture are often blamed on climate change or other environmental variables, close analysis of the events always reveals some form of human cause coupled with an environmental stress^6^. Attributing cause to environmental variables ignores the important human dimension to disaster and can deflect responsibility and accountability^38^. Second, these disaster risks are often introduced and can increase in frequency and scale when dependent on technology and infrastructure to produce in environments not naturally conducive to the scale of production^26^. There is, then, a paradox where the increasing sophistication of systems of production can lead to greater risk of disasters, a concept termed the manufacturing of risk^39^. The third and final point is that disasters are often a consequence of economic systems that are shaped by intense competition, financialization of industry, and a lack of regulatory oversight, since these processes can rush development while reducing emphasis on risk assessment^39^. The attribution to natural factors, increased reliance on technology, and increasingly competitive industry are characteristics of global salmon aquaculture.

While we propose that global salmon aquaculture may be an industry prone to these disasters because of the above points, our analyses cannot confirm this. We instead wish to raise these questions and open new inquiry into salmon aquaculture through the lens of MME risks, their causes, how they are responded to, and the extent to which they are becoming normalized in this important sector of the marine economy. Our analysis suggests that MMEs have grown and therefore we propose greater attention to the implication of these events. In particular, we suggest that salmon aquaculture should face questions raised in other sectors about the risks of optimizing production in systems of environmental and biology variability, and the risks of relying on these production systems.

## Methods

### Data collection

To explore the global scope of MMEs, we collected data on salmon mortality from the world’s major salmon aquaculture nations (Norway, Canada, the UK, Chile, Australia, New Zealand). First, we mapped the events spatially to determine the geography of MMEs. Next, we examine whether the frequency and scope of MME loss has changed over time (in particular, we look to see if the scope of loss of individual MMEs has changed over time). Finally, we use extreme value theory (EVT) to explore the estimated maximum potential loss of an event per country, comparing it to known estimates of specific company (or county) thresholds of loss.

Salmon mass mortality data were collected using an extensive literature search (through Web of Science, Scopus, Google Scholar, Google Books, SciFinder, Engineering Village, ResearchGate, Semantic Scholar, JSTOR, and Aquatic Sciences and Fisheries Abstracts), as well as aquaculture companies’ websites and annual production reports, data published in newspapers and magazines, and government websites. In cases where data was not accessible using these resources, and to vet our data in cases where it was available, we established contacts with relevant government departments. We requested them to share salmon mass mortality data (see Appendix for data sources associated with each recorded MME event).

Our literature search used diverse search terms about MMEs. Definitions of “mass mortality” vary by each jurisdiction. Some regions use the term “fish die-off” instead of MME, and some use “fish kill”. Some countries have a defined MME term, with many definitions focused on the rate of loss in a production site, but sometimes focused on rates of absolute loss (that is, kg of fish loss over a given time period^24^). For example, in British Columbia, Canada, MMEs are defined either by an absolute loss of 4000 kg over a day or a loss of 10,000 kg over 5 days. They can also be defined by relative rate measures, such as if 2% of current stock inventory lost over a day or 5% lost over 5 days. In Norway, MMEs are defined by daily relative rates of loss per fish cage rather than total production site inventory. In Scotland, MMEs are defined either by daily or monthly rates of loss per site. Individual companies and production regions (such as counties in Norway) can also develop their own definitions of MMEs, and the definition of MME can vary from company to company. For example, in Chile, most companies consider MME if the combined annual loss surpasses a certain threshold. In many countries, companies only report their loss events as MME if their annual loss exceeds a specific threshold, which is often some percentage of fish harvested and varies from company to company.

Because of the different definitions around the world and at different scales, and the different reporting standards from each country (see below), we did not analyze mortality against a common standard. Instead, within each country we analyzed the trends in mortality for each country, which allows us to track trends specific to each country’s reporting standards. Within each country, we focus on tracking the most severe loss events (as recorded by each country’s reporting standards) over time and explore if these are increasing in frequency and magnitude. For example, in Canada most events are reported at monthly or lower intervals at the production site scale, while in New Zealand mortality is reported at the scale of production site, but mortality is reported yearly. In isolating the most severe cases we can remain agnostic on a precise global definition of MME while simultaneously tracking the most severe loss events at a country level, therefore providing some quantitative evidence behind potential trends in MMEs around the world.

To explore mortality data relevant to country-specific metrics of concern for MMEs, we collected data of reported threshold values within individual companies in individual countries. In Norway, where data on loss is reported at county scales and not at scales of the production site, we collected data on county-defined scales of thresholds. We assumed that the reported thresholds from companies (or counties, for Norway) represent either a capacity to manage and respond to MMEs and/or a level of concern for animal welfare and disease spread, which allowed us to compare potential loss against capacity to manage MMEs. The available data on these thresholds is not a random sample nor a comprehensive sample (not all companies or counties publish this), so we use these measures in an illustrative rather than a conclusive study (see Supplemental data for threshold values by country).

We found that Canada and the UK report MMEs on a scale of production site per month or less (indicating limited aggregation and even per-event scale), Norway reports monthly mortality per county, while Chile, Australia, and New Zealand report losses at yearly intervals. The data for these latter three countries are therefore highly aggregated and difficult to analyze for trends. Yearly statistics leads to an order of magnitude less data to analyze for trends compared to monthly data, considerably decreasing the statistical power of the analyses conducted. We therefore analyze the data differently for the countries (described below). For Norway, we also collected data on the number of production sites during the time we have mortality data in each county and across the country (2012–2022). By analyzing trends in production sites we can determine whether trends in MMEs (if they exist) follow trends in production sites (if they exist) or not.

### Data analysis

From the data collection, we were first able to map out the frequency and magnitude of mortality. Using georeferenced data, we developed a spatial map of mortality around the world over time. We used the Flourish® software to develop a dynamic map showing where MMEs have occurred over time (using the Flourish® software—https://flourish.studio/).

To explore the temporal trends in MMEs we explore both the frequency and the magnitude of events over time. First, to analyze if the trend of MMEs is increasing in frequency, we isolated the top 10% of recorded events as measured by the size of the loss per recorded event within each country. These events were reflected to track the most severe loss events recorded within each country (Supplementary Table 1). We then tracked when these events occurred and if the frequency of occurrence had grown over time. In isolating the top 10% of cases we can remain agnostic on the precise definition of MME while simultaneously tracking the most severe loss events, at a country level according to the standards of reporting mortality within each country. Because some countries only record loss at yearly intervals, this leads to significant aggregations of data and loss in statistical power. In some cases (e.g. New Zealand and Australia), there are only 10 recorded events each, meaning that choosing the top 10% would not allow for any consideration of trends. Therefore, for countries where data is recorded on a yearly time-step, we chose to track the frequency of the top 50% of cases. We explore these countries for illustrative purposes and note that because of their data aggregation, conclusive statements on trends of MMEs cannot be made.

Because data collection ended during 2022, we did not capture all recorded MMEs for 2022 and in some nations, even MMEs for 2021 because of lags in data recording. For example, our database includes details of only three MMEs from Canada in 2021, but a recent search of the public database in Canada shows 85 events in 2021 in British Columbia alone (https://www.pac.dfo-mpo.gc.ca/aquaculture/reporting-rapports/episodes-mort-events/index-eng.html). However, the number of fish killed in each of these 85 events is not available. Therefore, for our analysis of frequency of the top 10% largest mortality events, we only use data for years we are confident in our database being comprehensive, which is from 2012–2021 for Norway, 2013–2020 for Canada, 2015–2021 for the UK, 2013–2021 for Chile, 2013–2021 for Australia, and 2013–2021 for New Zealand.

Second, we explored the magnitude of events over time. While the data is highly variable and the processes that generate the data (human decisions combined with technological innovation and environmental variation) make precise future prediction difficult or even impossible, we focus on analyzing trends within the time period of the database. In doing so we are not interested in developing inferential or predictive models but rather describing how change has occurred in the scope of mass mortalities over the database of events. We are interested in how the scale of the largest losses have changed over time, and so track the upper limit of magnitude of loss events. We analyzed the change in the upper bound of loss over time by calculating the maximum value of MMEs (as measured by magnitude of loss in numbers of fish) in each time period (monthly or yearly, depending on the country).

For tracking trends in both frequency and upper bound magnitude of loss events over time, we conducted a non-parametric Mann–Kendall tests to see if there are monotonic trends and used local polynomial regressions (lowess regression) to visualize the trends over time^40^. Though some evidence suggests that mortality in salmon aquaculture can follow seasonal patterns^5^, and so conducting a seasonal Mann–Kendall test may more accurately account for seasonal trends^41^, we chose to not to conduct a seasonal test, in order to utilize a more conservative test in the face of potential seasonal patterns. That is, should seasonal patterns be present in the data, a non-seasonal Mann–Kendall test would face more unexplained variation than a seasonal test and therefore be less likely to find significant trends. So, our decision for the type of test employed does not presuppose patterns and also is conservative against potentially finding patterns that are not real should random variation resemble seasonal shifts (i.e. we are less likely to commit type I errors). We only conducted these tests on countries with monthly or shorter intervals of data reporting, because of the issues of data aggregation and low sample sizes addressed above. With lowess regression we explored frequency at monthly and yearly intervals. While monthly scale analysis can provide finer resolution, it can also add noise to trends, especially if there are seasonal or other sub-annual patterns in the data, so a yearly trend of frequency was also considered to provide a more general indication of trends in the frequency of MMEs.

Because the Mann–Kendall test assumes independence, we tested for autocorrelation in our data by testing for correlations between each time period against subsequent lag time MMEs. Where autocorrelation was found, we first averaged our data across the time period where data is autocorrelated, ensured that autocorrelation was no longer present, then ran our analysis on the resulting averaged data. We found little to no evidence for autocorrelation in our data on maximum MMEs per time-period, globally and within countries, though we did find evidence for autocorrelation in determining a trend in the number of MMEs over time and adjusted accordingly before conducting statistical analyses of trends. For all Mann–Kendall tests we relied on 2-tail tests with a significance threshold α = 0.05.

Finally, we focused on understanding the extent of losses from individual MMEs. First, we describe the distribution of known MMEs per country, then generate estimates of the maximum loss of salmon that could occur. Here again we differentiate between Norway, Canada, and the UK on one hand (where loss at monthly or lower resolutions is available) and Chile, Australia, and New Zealand on the other hand (where loss per year is available). In this way we estimate the maximum potential loss per event in the former set of countries and the maximum potential loss per year in the latter set of countries.

To estimate the maximum potential loss of salmon within each country and to compare against what each country defines as an MME, we relied on EVT. EVT focussed on understanding the behavior of maxima or minima^42,43^. Therefore, we used EVT to compute the maximum loss at the distribution tail. EVT employs two main strands of models: Peak Over Threshold (POT) and Block Maxima (BM)^43^. The block maxima method only considers the maximum observations for each non-overlapping, equal-sized interval of the observation period. The POT method is thought to be more data efficient because it makes better use of all available information and is thus mostly used for practical applications, and is frequently used in risk management^43^. In applying EVT, we use the POT approach and fit the resulting data to the generalized Pareto distribution (GPD) distribution. As a threshold, we assessed loss of the top 10% of events as measured by the number of fish lost for Norway, Canada, and the UK. For Chile, Australia, and New Zealand we used the top 50% of cases as the threshold. In this way, we treated MMEs consistently in estimating the maximum size of events within each country and in tracking MME frequency and scale over time (see previous methods on time series).

We used Value at Risk (VaR) and Expected Shortfall (ES) analysis to assess the maximum expected salmon loss in an MME in a country. VaR calculates the worst loss possibly occurring in the given time frame and at a given confidence level^43^. In other words, VaR shows the maximum loss that one can expect with a given confidence level. Here, we focused on a 99.9% confidence level for illustration purposes. While VaR is suitable for assessing the maximum salmon loss, VaR cannot estimate the quantity of loss above the given confidence level.

On the other hand, ES (sometimes called conditional VaR) assesses the quantity of loss above this confidence level and is calculated from VaR^44^. ES is often a preferred risk assessment tool when facing highly variable data (such as the case of salmon mass mortalities)^43^. So, if VaR can be used to estimate the maximum number of fish that can be expected to be lost at the 99.9 percentile worst case, ES can estimate the expected potential loss within the worst 0.1% cases. For Norway, Canada, and the UK, we compare these estimates against the average mortality event threshold to compare an estimated worst case event against the capacity to address mortality events. For Chile, Australia, and New Zealand, we compare the estimated yearly potential maximum loss against the yearly threshold. We therefore calculated the ES as the expected loss within the worst 0.1% of cases, and when plotting the results included error bars representing the point estimate of the worst 0.1% event (as the lower bound) and the worst 0.01% event (as the upper bound).

### Supplementary Information

Supplementary Information 1.

Supplementary Information 2.

## Data Availability

All data generated or analysed during this study are included in this published article (and its Supplementary Information files).

## References

[1] Gephart JA, Golden CD, Asche F, Belton B, Brugere C, Froehlich HE, Fry JP, Halpern BS, Hicks CC, Jones RC, Klinger DH, Little DC, McCauley DJ, Thilsted S (2021). Scenarios for global aquaculture and its role in human nutrition. Rev. Fish. Sci. Aquac..

[2] Naylor R, Fang S, Fanzo J (2023). A global view of aquaculture policy. Food Policy.

[3] GSI. *Sustainable Salmon Farming: The Future of Food* 33 (Global Salmon Initiative, 2021). https://globalsalmoninitiative.org/files/documents/GSI_Handbook_2020.pdf.

[4] Aunsmo A (2023). Real-time monitoring of cause-specific mortality- and losses in industrial salmon farming. Aquaculture.

[5] Bang Jensen, B., Qviller, L. & Toft, N. Spatio‐temporal variations in mortality during the seawater production phase of Atlantic salmon (*Salmo salar.*) in Norway. *J. Fish Dis.* **43**, 445–457 (2020). 10.1111/jfd.13142 32057123

[6] Oliveira VHS, Dean KR, Qviller L, Kirkeby C, Bang Jensen B (2021). Factors associated with baseline mortality in Norwegian Atlantic salmon farming. Sci. Rep..

[7] Persson D, Nødtvedt A, Aunsmo A, Stormoen M (2022). Analysing mortality patterns in salmon farming using daily cage registrations. J. Fish Dis..

[8] Pincinato RBM, Asche F, Bleie H, Skrudland A, Stormoen M (2021). Factors influencing production loss in salmonid farming. Aquaculture.

[9] Wheatley, S., McLaughlin, M., Menzies, F. & Goodall, E. Site management factors influencing mortality rates in Atlantic salmon (*Salvo**salar* L.) during marine production. *Aquaculture* **136**, 195–207 (1995).

[10] Sommerset, I. *et al*. *Norwegian Fish Health Report 2021, Norwegian Veterinary Institute Report, series #2a/2022* (Norwegian Veterinary Institute, 2022).

[11] Fisheries and Marine Institute. *A Review of the 2019 Newfoundland and Labrador South Coast Cultured Atlantic Salmon Mortality Event*. https://www.gov.nl.ca/ffa/files/publications-pdf-2019-salmon-review-final-report.pdf (2020).

[12] Martin J (2006). Salmon mortalities associated with a bloom of *Alexandrium fundyense* in 2003 in the Bay of Fundy, and subsequent early warning approaches for industry. Afr. J. Mar. Sci..

[13] Saksida S (2006). Infectious haematopoietic necrosis epidemic (2001 to 2003) in farmed Atlantic salmon Salmo salar in British Columbia. Dis. Aquat. Org..

[14] Bruno, D. W., Dear, G. & Seaton, D. D. Mortality Associated with Phytoplankton Blooms among Farmed Atlantic Salmon, *Salmo**salar *L., in Scotland. *Aquaculture* **78**, 217–222 (1989).

[15] Cronin, M. *et al. Salmon mortalities at Inver Bay and McSwyne’s Bay Finfish Farms, County Donegal, Ireland, During 2003.* (2004).

[16] Armijo J, Oerder V, Auger P-A, Bravo A, Molina E (2020). The 2016 red tide crisis in southern Chile: Possible influence of the mass oceanic dumping of dead salmon. Mar. Pollut. Bull..

[17] Navedo JG, Vargas-Chacoff L (2021). Salmon aquaculture threatens Patagonia. Science.

[18] Mitchell, J. Authorities Investigating Mass Salmon Die-Off At Down East Aquaculture Operation. *Maine Public* https://www.mainepublic.org/business-and-economy/2021-08-27/authorities-investigating-mass-salmon-die-off-at-down-east-aquaculture-operation (2021).

[19] McDonagh, V. NZ King Salmon hit by heavy mortalities: Fish Farmer Magazine. *Fish Farmer Magazine* (2022).

[20] Asche F, Roll KH, Sandvold HN, Sørvig A, Zhang D (2013). Salmon aquaculture: Larger companies and increased production. Aquac. Econ. Manag..

[21] Afewerki S, Asche F, Misund B, Thorvaldsen T, Tveteras R (2023). Innovation in the Norwegian aquaculture industry. Rev. Aquac..

[22] Gamperl, A. K., Caballero-Solares, A., Lehnert, S. J. & Filgueira, R. *Mass Mortalities at Marine Salmon Cage-Sites: Environmental Factors, Biological Phenomena, and Their Interactions* (2022).

[23] Romero J, Feijoó CG, Navarrete P (2021). Antibiotics in aquaculture–use, abuse and alternatives. Health Environ. Aquac..

[24] Neis B (2023). Mass mortality events in marine salmon aquaculture and their influence on occupational health and safety hazards and risk of injury. Aquaculture.

[25] Souto Cavalli L, Tapia-Jopia C, Ochs C, López Gómez MA, Neis B (2023). Salmon mass mortality events and occupational health and safety in Chilean aquaculture. All Life.

[26] Giddens A (1999). Risk and responsibility. Mod. Law Rev..

[27] Knowles S (2014). Learning from disaster?: The history of technology and the future of disaster research. Technol. Cult..

[28] Hvas M, Folkedal O, Oppedal F (2021). Fish welfare in offshore salmon aquaculture. Rev. Aquac..

[29] FAO. *Fisheries and Aquaculture: Fisheries and Aquaculture—Statistics*. https://www.fao.org/fishery/en/statistics (2023).

[30] Sumaila UR (2022). Aquaculture over-optimism?. Front. Mar. Sci..

[31] Just Economics. *Dead Loss: High Cost of Poor Farming Practices and Mortalities on Salmon Farms*. https://www.justeconomics.co.uk/health-and-well-being/dead-loss (2021).

[32] Mascareño A (2018). Controversies in social-ecological systems: Lessons from a major red tide crisis on Chiloe Island, Chile. E&S.

[33] Billing S-L (2018). Using public comments to gauge social licence to operate for finfish aquaculture: Lessons from Scotland. Ocean Coast. Manag..

[34] Antonucci F, Costa C (2020). Precision aquaculture: A short review on engineering innovations. Aquacult. Int..

[35] Føre M (2018). Precision fish farming: A new framework to improve production in aquaculture. Biosyst. Eng..

[36] O’Donncha F, Grant J (2019). Precision aquaculture. IEEE Internet Things M..

[37] Rastegari H (2023). Internet of Things in aquaculture: A review of the challenges and potential solutions based on current and future trends. Smart Agric. Technol..

[38] Ribot J (2022). Violent silence: Framing out social causes of climate-related crises. J. Peasant Stud..

[39] Watts M, Soederberg S (2010). Accumulating insecurity and manufacturing risk along the energy frontier. Research in Political Economy.

[40] Hirsch R, Slack JR, Smith RA (1982). Techniques of trend analysis for monthly water quality data. Water Resour. Res..

[41] Ha D-W (2022). Long-term water quality fluctuations in the Seomjin river system determined using LOWESS and seasonal Kendall analyses. Water Air Soil Pollut..

[42] Pratiwi N, Iswahyudi C, Safitri RI (2019). Generalized extreme value distribution for value at risk analysis on gold price. J. Phys. Conf. Ser..

[43] Aleksandra Brdar T, Nota G (2010). Quantitative operational risk management. Advances in Risk Management.

[44] Embrechts P, Furrer H, Kaufmann R, Mikosch T, Kreiß J-P, Davis RA, Andersen TG (2009). Different kinds of risk. Handbook of Financial Time Series.

