# Supplementary material for: Quantitative analysis of mass mortality events in salmon aquaculture shows increasing scale of fish loss events around the world
Source: Sci Rep. 2024 Mar 7;14:3763. doi: 10.1038/s41598-024-54033-9 (PMC10920753; doi:10.1038/s41598-024-54033-9)
Supplement: Supplementary file 1 — Supplementary Information 1. [file 41598_2024_54033_MOESM1_ESM.docx]

**Supplement**

**Supplementary Methods**

**Extreme Value Analysis**

Extreme value theory (EVT) focuses on understanding the behavior of maxima or minima. In this study, we are interested in using EVT to estimate how big an MME could occur. Therefore, we will use EVT to compute the maximum loss at the distribution tail. To analyze the behavior of distributions' tails, EVT employs two main strands of models: Peak Over Threshold (POT) and Block Maxima (BM). The block maxima method only considers the maximum observations for each non-overlapping, equal-sized interval of the observation period. The generalized extreme value (GEV) distribution is followed by collecting extreme observations chosen under extreme value criteria. Peak-over-threshold (POT) methodology chooses extreme observations that surpass a high threshold and, for this reason, is chosen in this study. A GPD closely resembles the probability distribution of exceedances over a high threshold. The POT method is thought to be more data efficient because it makes better use of all available information and is thus mostly used for practical applications. EVT examines improbable situations with potentially disastrous consequences. EVT focuses on the estimation of extreme risk as well as the modeling of the limiting distributions produced by the tail distribution of the extreme values. The POT method is frequently employed methods in risk management ^39^. The POT technique considers the distribution of exceedances (F) across a specific high threshold (u), which is defined by ^38^

F_u_(y) = Pr(X-u≦y|X>u) = $\frac{F\left( y+u \right)-F(u)}{1-F(u)}$, where 0≦y≦x_F_ – u

According to the Gnedenko-Pickands-Balkema de Haan Theorem^40^, for a large dataset of distribution function (F), and for an increasing u, the excess conditional distribution function Fu(y) can be computed by

F_u_(y) ≈ G_ζ, σ_ (y), u →∞

G_ζ, σ_ (y) is GPD and is defined as

G_ζ, σ_ (y) = $\{1-J 1-Q$

Where J = ${(1+\frac{\zeta}{\sigma}y)}^{-1/\zeta}$ when 𝜁≠0, and

Q = -exp(-y/σ) when 𝜁 = 0

In the GPD equation above, y ∈ [0, (x_F_ – u)], if 𝜁≥0 and, y ∈[0, -σ/𝜁] if 𝜁<0.

In the GPD distribution, σ is the scale parameter, and 𝜁 is called the shape parameter of the distribution. Based on the shape factor, GPD can have a heavy, light, or short tail based on the following conditions.

GPD has heavy-tailed Pareto distribution if 𝜁>0

GPD has a short-tailed Pareto distribution if 𝜁<0

GPD has a light-tailed exponential distribution if 𝜁 = 0

Kurtosis of distribution of each country dataset is performed to find out the shape (heavy, light or short-tailed) of the distribution. The following formula is used to compute the kurtosis of each country MME data.

Kurtosis = $\frac{\sum{(xi-\underline{x)}}^{4}}{n\sigma^{4}}$

Where x is the mean of the MME data and σ is its standard deviation. Following formulas are used to compute the mean and standard deviation.

$\underline{x}$ = $\frac{\sum xi}{n}$ and σ = $\sqrt{\frac{{\sum(xi- \underline{x})}^{2}}{n}}$

Where n is the number of data points.

The calculation procedure for Chile is shown below and is the same for the rest of the countries.

| Mortality x10^6 (xi) | (x-x)^2^ | (x-x)^4^ |
| --- | --- | --- |
| 23.0000 | 459.8214 | 211435.7116 |
| 4.5489 | 8.9541 | 80.1754 |
| 4.0480 | 6.2073 | 38.5306 |
| 4.0046 | 5.9927 | 35.9126 |
| 3.9074 | 5.5267 | 30.5443 |
| - | - | - |
| - | - | - |
| - | - | - |
| 0.3000 | 1.5789 | 2.4930 |
| 0.3000 | 1.5789 | 2.4930 |
| 0.1490 | 1.9812 | 3.9252 |
| 0.1099 | 2.0928 | 4.3800 |
| 0.0200 | 2.3610 | 5.5743 |
| 0.0100 | 2.3918 | 5.7208 |
|  | ∑ = 557.1384 | ∑ = 211708.2085 |
|  |  |  |
| x= 1.5566 | σ = 2.1028 | Kurtosis = 85.9374 |

As indicated, kurtosis is more than 3, which shows that the distribution of Chile MME follows a heavy tail ^41^. Performing the above analysis on data from the rest of the countries shows that distribution of all countries’ data has a heavy tail.  This indicates that their profiles have 𝜁>0 for GPD.

The POT method application necessitates the determination of an appropriate threshold value, u. The threshold value is usually chosen as a compromise between variance and bias. The threshold u should ideally be set sufficiently high to ensure that exceedances have a limiting distribution that falls within the domain of attraction of GPD. In contrast, if u is set extremely high, there is a possibility of having too few exceedances to estimate the GPD parameters accurately. It is common to practice selecting a threshold value that is as low as possible while still providing a reliable asymptotic approximation of the limiting distribution. The threshold u is determined in this paper using two popular techniques: the mean excess function (MEF) ^42^ and the Hill estimator^431^.

Both parameters of GPD are estimated by maximizing the likelihood function at the value of u and are given by;

L(𝜁, σ|y) = $\{-nlog\sigma-(\frac{1}{\zeta}+1)\sum_{i=1}^{n} loglog \left( 1+\frac{\zeta}{\sigma}yi \right) , if \zeta\neq0 -nlog\sigma-(\frac{1}{\sigma})\sum_{i=1}^{n} yi, if \zeta=0$

Tails of GPD are estimated using results of exceedance distribution and through substituting F_u_(y) with G_ζ, σ_ (y) and substituting F(u) with the empirical estimator (1-N_u_)/n as shown below.

F(x) = (1-F(u)) G_ζ, σ_ (x-u) + F(u), valid for x>u

Therefore, the cumulative distribution function (CDF) for tail of GPD is as below

$\hat{F(x)}$ = N_u_/n (1- ${(1+\frac{\hat{\zeta}}{\hat{\sigma}}(x-u)}^{\frac{-1}{\hat{\zeta}}})$ + (1- N_u_/n)

$\hat{F(x)}$ = 1 - N_u_/n${(1+\frac{\hat{\zeta}}{\hat{\sigma}}(x-u)}^{\frac{-1}{\hat{\zeta}}})$

Therefore, Value at Risk (VaR) at a given probability p≥F(u), is estimated using.

VaR = u + $\frac{\hat{\sigma}}{\hat{\zeta}}{((n/Nu(1-p)}^{-\hat{\zeta}}-1)$

Where n is the number of observations in the tail end, and Nu is the number of excesses beyond the threshold value u.

Considering this, the expected shortfall (ES) or Conditional VaR is defined as

ES = VaR + $\frac{\sigma+\zeta(VaR-u)}{1-\zeta}$

ES = $\frac{VaR}{1-\zeta}$ + $\frac{\sigma+\zeta u}{1-\zeta}$

ES = $\frac{VaR+ \sigma+\zeta u}{1-\zeta}$

**Supplementary Figures**

<https://public.flourish.studio/visualisation/13258915/>

Supplementary Figure 1. Dynamic map of salmon MMEs around the world. The scale of events is in the number of salmon killed.


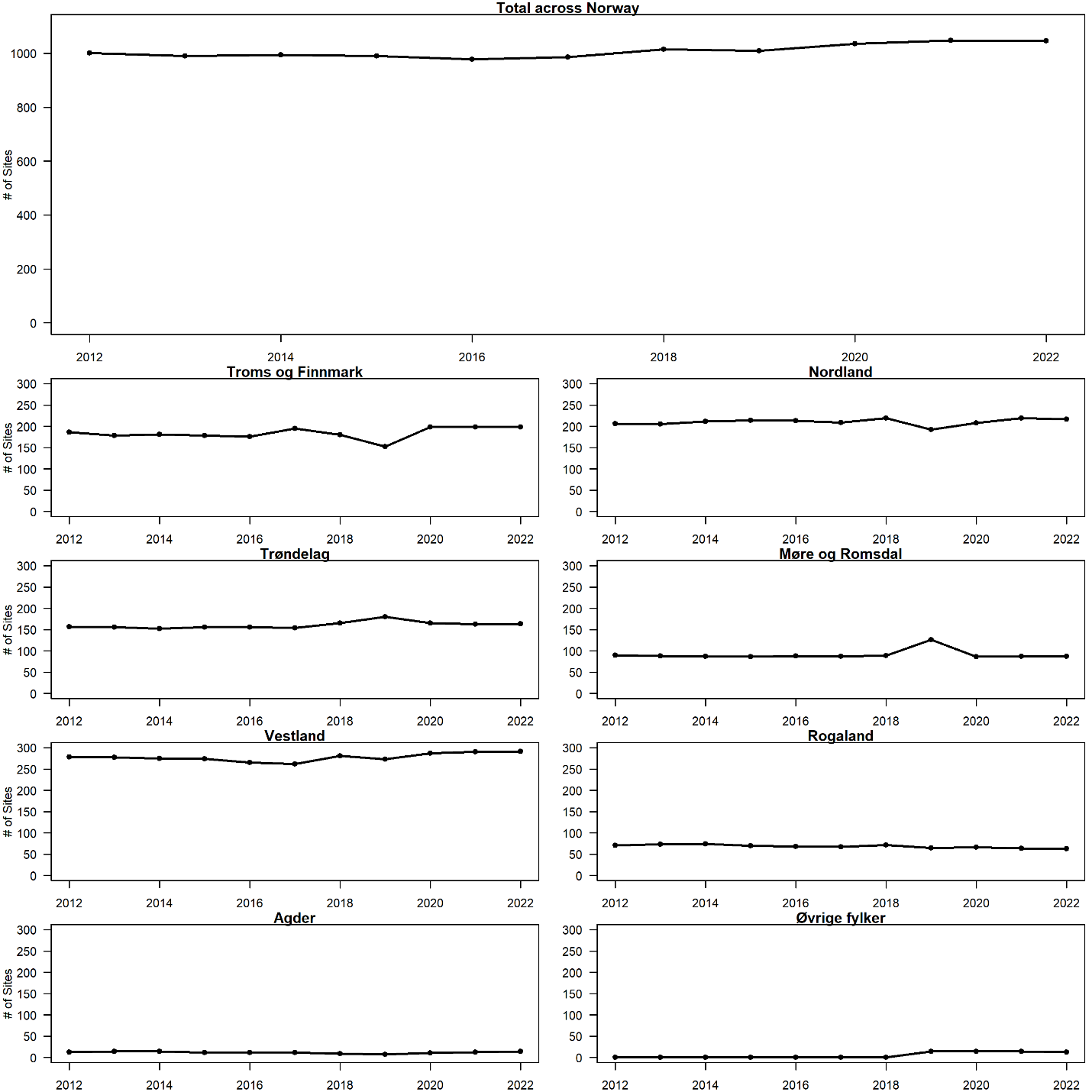


Supplementary Figure 2: Time series of production sites in Norway. No statistically detectable trends were observed for all sites across Norway (main figure, Kendall’s tau = 0.4, p= 0.462). Across the counties the number of sites declined between 2012-2017, then increased between 2018-2022 (the number of sites ranged from 978-1047). No significant trends were observed across the individual reporting counties except for two, and these trends offset each other. For Toms og Finnmark no significant trend was observed (Kendall’s tau = 0.283, p=0.269). For Nordland no significant trend was observed (Kendall’s tau = 0.294, p=0.241). For Trøndelag no significant trend was observed (Kendall’s tau = 0.359, p=0.155). For Møre og Romsdal no significant trend was observed (Kendall’s tau = -0.177, p=0.520). For Vestland no significant trend was observed (Kendall’s tau = 0.273, p=0.276). For Rogaland a significant decreasing trend was observed (Kendall’s tau = -0.709, p=0.00309). For Agder no significant trend was observed (Kendall’s tau = -0.234, p=0.379). For Øvrige fylker a significant increasing trend was observed (Kendall’s tau = 0.605, p=0.0265). Øvrige fylker increase was entirely in land-based production sites, which overall an order of magnitude less production than other counties.


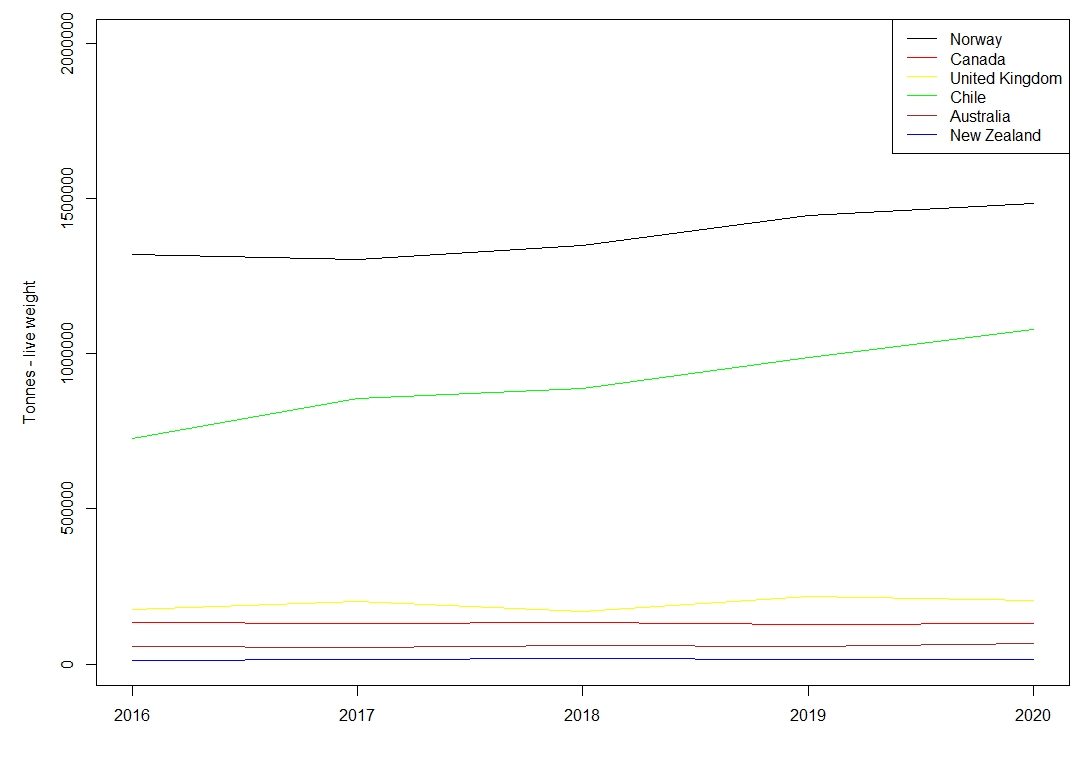


Supplementary Figure 3. Yearly production of salmon aquaculture by country. Data obtained from FAO

Supplementary Table 1. The thresholds (measured by number of fish killed) and number of events that make up the top 10% (or 50% in countries with yearly reporting) of mortality events within each country used in the temporal analysis of mass mortality events. Quantitative analysis was performed up to 2021 (2020 for Canada) due to data limitations on later years.

|  |  | Norway | Canada | United Kingdom | Chile* | Australia* | New Zealand* |
| --- | --- | --- | --- | --- | --- | --- | --- |
|  | Range of number of fish killed in top 10% (50% *) of events | 935205 - 4913023 | 154978 - 3830225 | 26035 - 1521479 | 1175556 - 4548889 | 792000 - 1335000 | 3659556 - 4170667 |
|  | Number of events within top 10% (50%*) | 97 | 537 | 374 | 61 | 5 | 4 |
| Number of events within the top 10% (50%*) by year | 2012 | 1 |  |  |  |  |  |
|  | 2013 | 4 | 32 |  | 8 | 1 |  |
|  | 2014 | 3 | 49 |  | 6 | 1 | 1 |
|  | 2015 | 6 | 79 |  | 7 | 0 | 1 |
|  | 2016 | 3 | 64 | 17 | 10 | 0 | 0 |
|  | 2017 | 5 | 42 | 64 | 4 | 1 | 0 |
|  | 2018 | 6 | 109 | 44 | 4 | 1 | 0 |
|  | 2019 | 8 | 86 | 73 | 7 | 1 | 0 |
|  | 2020 | 22 | 74 | 55 | 8 | 0 | 1 |
|  | 2021 | 22 | 2 | 96 | 7 | 0 | 1 |
|  | 2022 | 17 |  | 25 |  |  |  |

* indicates where analysis was done on the top 50% of events rather than top 10%. Where cells in the table are missing numbers indicates dates of data paucity.

**Literature Cited**

1. Aleksandra Brdar, T. Quantitative operational risk management. in *Advances in Risk Management* (ed. Nota, G.) (Sciyo, 2010). doi:10.5772/270.

2. Pratiwi, N., Iswahyudi, C. & Safitri, R. I. Generalized extreme value distribution for value at risk analysis on gold price. *J. Phys.: Conf. Ser.* **1217**, 012090 (2019).

3. Omari, C. O., Mwita, P. N. & Waititu, A. G. Using Conditional Extreme Value Theory to Estimate Value-at-Risk for Daily Currency Exchange Rates. *JMF* **07**, 846–870 (2017).

4. Kallner, A. *Laboratory statistics*. (Elsevier, 2017).

5. Ghosh, S. & Resnick, S. A discussion on mean excess plots. *Stochastic Processes and their Applications* **120**, 1492–1517 (2010).

6. Knight, K. A simple modiﬁcation of the Hill estimator with applications to robustness and bias reduction. (2007).
